# A Ventriculoperitoneal Shunt With Anal Protrusion Causing Meningitis in A Child

**DOI:** 10.7759/cureus.45857

**Published:** 2023-09-24

**Authors:** Ahmed Basehi, Abdullah M Al-saleh, Haitham Almoffarreh, Sarmad Alkarawi, Mohammed Alharbi

**Affiliations:** 1 Pediatric Emergency Medicine Department, Ministry of the National Guard – Health Affairs, Riyadh, SAU; 2 Pediatric Emergency Medicine Department, King Abdullah International Medical Research Center, Riyadh, SAU; 3 Pediatric Emergency Medicine Department, King Saud Bin Abdulaziz University for Health Sciences, Riyadh, SAU; 4 Pediatric Neurosurgery Department, Ministry of the National Guard – Health Affairs, Riyadh, SAU; 5 Pediatric Neurosurgery Department, King Abdullah International Medical Research Center, Riyadh, SAU; 6 Pediatric Neurosurgery Department, King Saud Bin Abdulaziz University for Health Sciences, Riyadh, SAU; 7 Pediatric Medicine Department, Maternal and Children Hospital, Qassim, SAU

**Keywords:** child, meningitis, complication, anal protrusion, ventriculoperitoneal shunt

## Abstract

The ventriculoperitoneal (VP) shunt has been one of the primary methods for treating hydrocephalus for many years and is one of the most frequent surgical interventions performed in neurosurgery using a variety of techniques and different VP shunt types. Consequently, shunt insertion is associated with many complications, including insertion failure, functional failure, and mechanical failure such as shunt migration. Shunt migration to the gastrointestinal or urogenital tract is a rare and one of the most distressing complications, which can lead to ascending infection and even meningitis. We report a rare case of a 24-month-old male with a VP shunt tube that migrated and protruded from the anus, subsequently causing meningitis.

## Introduction

A ventriculoperitoneal (VP) shunt is a medical device used to treat hydrocephalus, a condition in which there is an excessive accumulation of cerebrospinal fluid (CSF) in the brain [1-3]. The shunt consists of a tube that is surgically implanted into the brain's ventricular system and another tube that leads from the shunt valve to the peritoneal cavity in the abdomen. Additionally, the VP shunt works by draining excess CSF from the brain's ventricles and transporting it to the peritoneal cavity, where it can be absorbed and eliminated by the body [4]. The shunt system typically includes a valve that regulates the flow of CSF and prevents over-drainage or under-drainage. VP shunts are usually placed under general anesthesia, and the surgery typically takes one to two hours [5-7].

After the procedure, patients may experience some discomfort and need to stay in the hospital for a few days. They will also need to have regular follow-up appointments with their healthcare provider to monitor the shunt's function and address any complications that may arise [8,9]. Therefore, it is possible for a VP shunt to cause complications, such as meningitis, if it is not functioning properly or if it becomes infected [10,11]. In the case of a child with an anal protrusion, this could potentially increase the risk of infection, because the bacteria from the anal area could migrate up the shunt and cause an infection in the brain or spinal cord [3-11].

## Case presentation

A 24-month-old male presented to the pediatric emergency department (ED) with a history of tube protruding from the anus. He had undergone a VP shunt at the age of six days for congenital hydrocephalus and was discharged home without any postoperative complications, followed by two shunt revisions at the age of 16 months and 17 months.

Seven months after the last shunt revision at the age of 17 months, the child presented to the pediatric ED with a distal catheter protruding through the anus; this was noticed by his mother one hour before presentation to ED.

**Figure 1:**
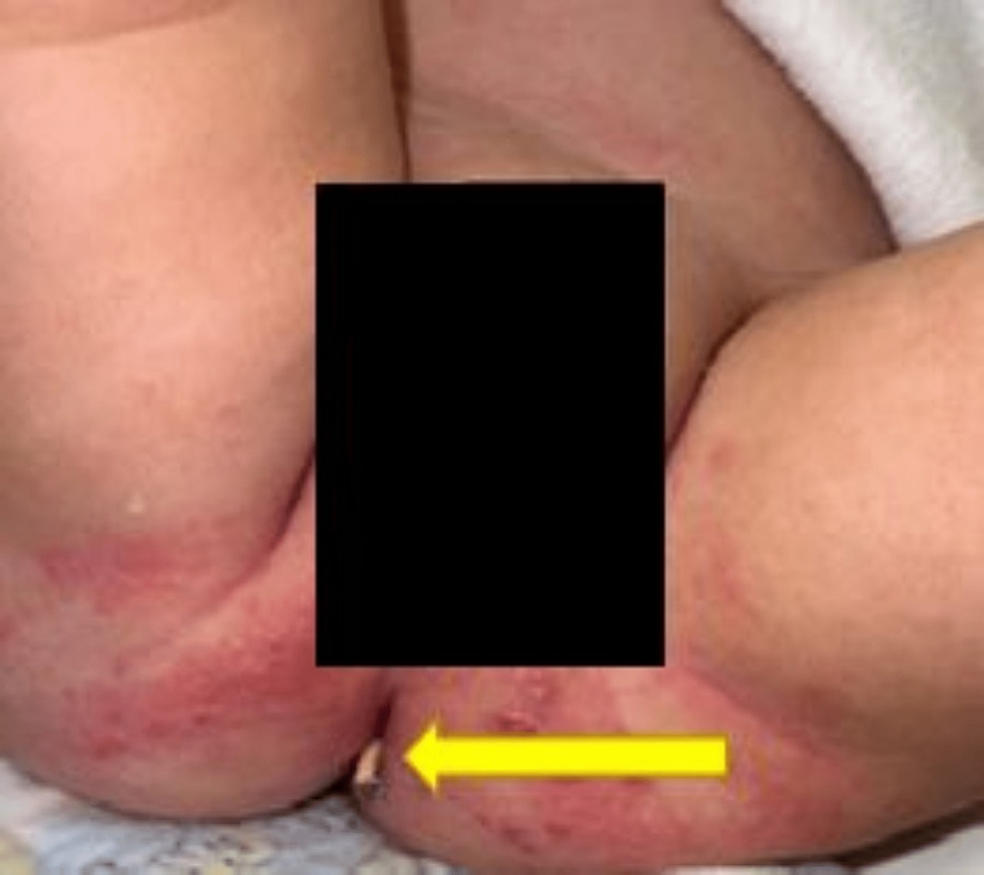
VP shunt protruding from the anus. This figure shows the VP shunt protruded out from the anus. VP: ventriculoperitoneal.

There was no history of fever, headache, vomiting, abnormal movements, or abdominal distention. Clinical examination did not reveal any signs of peritonitis or meningitis. The peritoneal end of the VP shunt was protruding through the anus (Figure 1).

There was dribbling of CSF at the distal end of the VP shunt. The child was investigated with a VP shunt survey X-ray, a computed tomography (CT) scan of the brain, and a full septic work-up that included a complete blood count, inflammatory markers, blood culture, and a CSF sample for CSF studies, cultures, and PCR. The patient was kept nil by mouth, and empirical antibiotics (ceftriaxone and vancomycin) were started.

**Figure 2:**
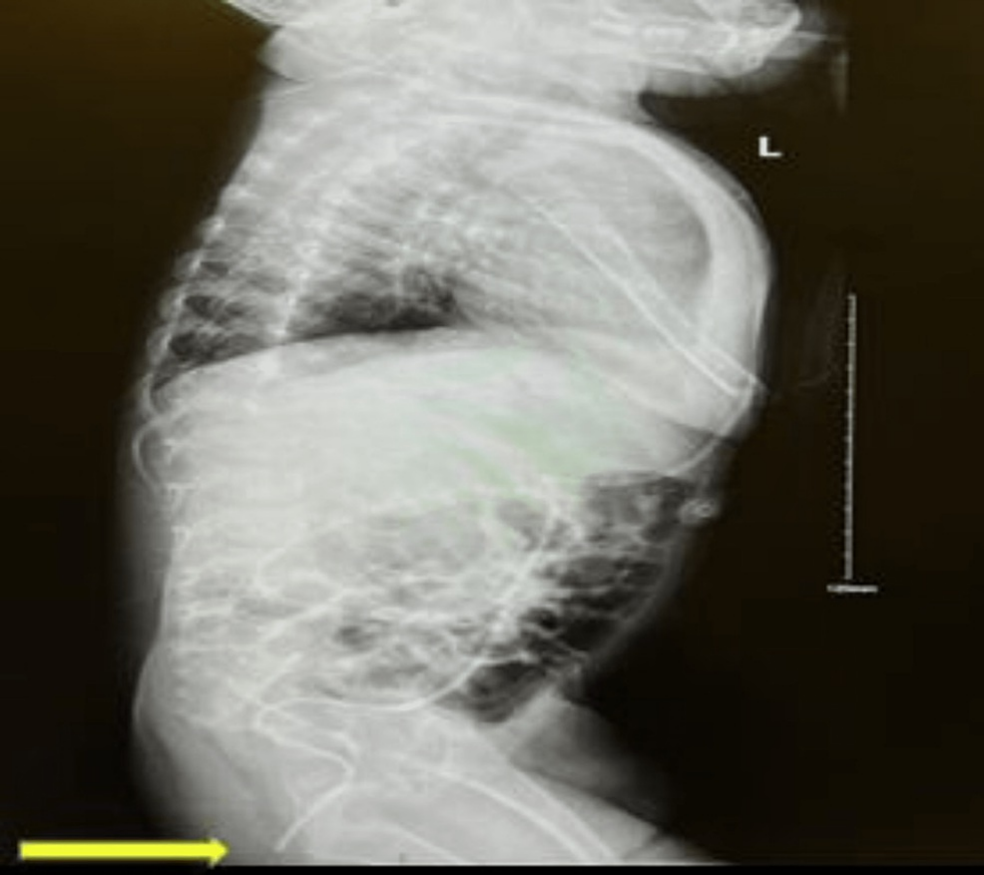
The VP shunt survey X-ray. The image is confirmed the peritoneal end of the shunt tube is going well beyond the pubic symphysis.

The VP shunt survey X-ray confirmed that the peritoneal end of the shunt tube was extending well beyond the pubic symphysis; no knotting of the shunt tube was visualized. No gas under the diaphragm was noted (Figure 2).

**Figure 3:**
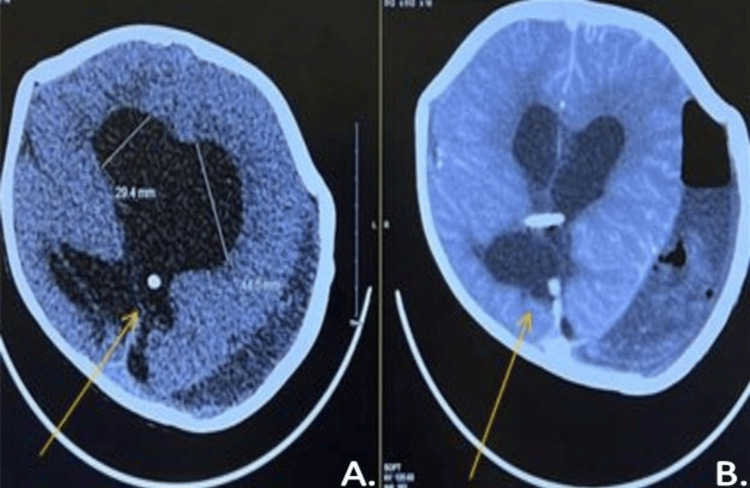
New and old brain CT. Comparison of the new brain CT (A) with the previous brain CT (B), which was done six months ago, shows a left frontal approach VP shunt with its tip seen at the occipital horn of the right lateral ventricle. There is an interval increase in the size of the supratentorial ventricular system.

CT scans of the brain, comparing the new brain CT (Figure 3A) to the previous brain CT (Figure 3B) performed six months ago, show a left frontal approach VP shunt with its tip seen at the occipital horn of the right lateral ventricle. There is an interval increase in the size of the supratentorial ventricular system (Figure 3A-3B).

CBC demonstrated a high white blood cell count (WBC), normal hemoglobin, and normal platelet levels; blood culture was negative. Inflammatory markers included high erythrocyte sedimentation rate (ESR), high C-reactive protein (CRP), and high procalcitonin. The CSF sample showed a high white blood cell count, high red blood cell (RBC) count, normal lymphocytes, high neutrophils, normal monocytes, high CSF protein levels, and low CSF glucose. The CSF culture grew Providencia stuartii. After identifying the CSF culture, the antibiotics vancomycin and ceftriaxone were upgraded to meropenem (Table 1).

**Table 1:** Cerebrospinal fluid and Inflammatory markers investigations result g/dL: grams per deciliter; mmol/L: millimoles per liter; mm/hr: millimeters per hour; mg/L: milligrams per liter; ng/mL: nanograms per milliliter; ESR: erythrocyte sedimentation rate; CRP: C-reactive protein; PCT: procalcitonin; WBC: white blood count; CSF: cerebrospinal fluid.

Examination Name	Result	Reference Value
Complete blood count		
Hemoglobin	137	110~145 g/L
White blood count (WBC)	12.10	4~12 x10^9^/L
Platelet	334	150~450 x10^9^/L
Cerebrospinal fluid data		
CSF color	Xanthocromia	Clear
CSF appear	Slightly cloudy	Clear
CSF WBC	450	0~5 x 10^6^/L
CSF RBC	72	0~10 x 10^6^/L
CSF neutrophil	41	0~6%
CSF monocyte	17	15~40%
CSF lymphocyte	42	40~80%
CSF protein	2.15	0.15~0.40 g/L
CSF glucose	1.40	3.3~4.5 mmol/L
CSF culture	Providencia stuar	Negative
Inflammatory markers		
Erythrocyte sedimentation rate (ESR)	40	0~15 mm/hr
C-reactive protein (CRP)	33	~8 mg/L
Procalcitonin (PCT)	0.11	~0.05 ng/mL

On Day 1 of admission, the patient was brought to the operating room for laparoscopic shunt removal, performed by pediatric surgery, and the insertion of a new external ventricular drainage, carried out by pediatric neurosurgery. In the first stage, the pediatric surgery team conducted a diagnostic laparoscopy, reporting multiple adhesions and a thick peritoneum. The site of the peritoneal shunt was facing the rectum but could not be identified due to the presence of multiple adhesions; the distal catheter was removed. No area of leakage or perforation was noted. The pelvis was found to be severely adhered. No other complications were observed.

In the second stage, pediatric neurosurgery reported that the VP shunt valve was exposed at the left frontal area. The skin was opened using the same previous skin incision around the valve in a curved fashion. The valve was fully exposed, and the distal tube was cut and pulled through the anus; it was found to be free. The valve, along with the proximal catheter, was removed and sent for culture, which grew Providencia stuartii. A new external ventricular drainage (EVD) catheter was placed and secured. The CSF was released under low pressure and was tunneled through the skin, then secured using 2-0 silk sutures. Dressing was applied, and the patient tolerated the procedure well. Postoperatively, the patient was extubated and transferred to the pediatric intensive care unit in a stable condition.

**Figure 4:**
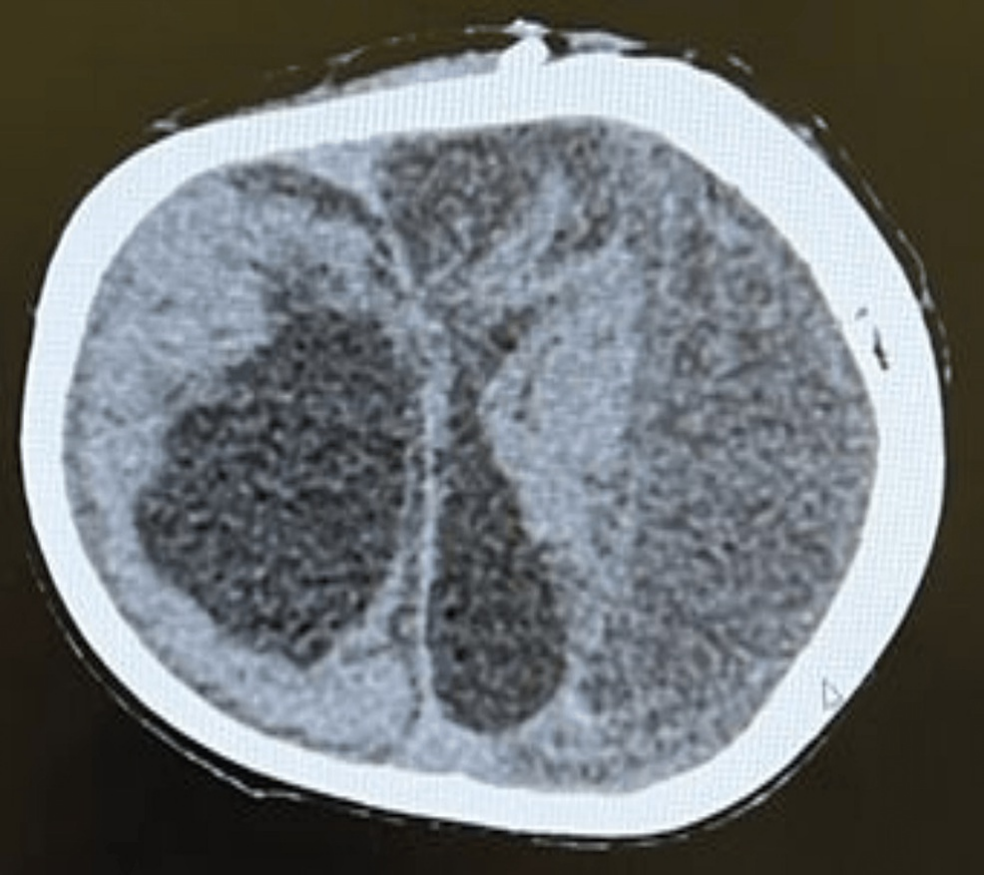
Repeated brain CT. The image reveals that the tip of the VP shunt is situated in the encysted hydrocephalus on the right side. It also indicates an interval decrease in the size of the supratentorial hydrocephalus. Additionally, the image shows an interval increase in the size of the left cerebral convexity subdural collection, along with the development of a small right cerebral convexity subdural collection.

Subsequent CT scans of the brain showed that the tip of the VP shunt was located in the encysted hydrocephalus on the right side, with an interval decrease in the size of the supratentorial hydrocephalus. There was also an interval increase in the size of the left cerebral convexity subdural collection, along with the development of a small right cerebral convexity subdural collection (Figure 4).

After day 2 postoperatively, the patient was shifted to the regular floor with follow-up by the infectious team and daily CSF study. During the same admission, following clearance of CSF, the patient underwent another surgery by both pediatric surgery and neurosurgery for removal of the EVD and insertion of a new VP shunt.

## Discussion

Hydrocephalus is a condition characterized by excessive accumulation of fluid in the brain, leading to increased intracranial pressure. The term "hydrocephalus" is derived from the Greek words "hydro," which means water, and "cephalus," which means head [12,13]. Shunt surgery is the most commonly employed treatment for hydrocephalus [14,15]. During this procedure, a small tube is inserted into the brain to drain the excess fluid and redirect it to another part of the body, such as the abdomen, pleural cavity, or heart [16].

There are several types of shunts available, including VP, thecoperitoneal, ventriculoatrial, and ventriculopleural shunts. Among these, VP shunts are the most commonly used for treating hydrocephalus [17].

However, shunt surgery is associated with many risks. The complication rate for VP shunts is reported to be between 24% and 47%. The most common complication is mechanical blockage of the shunt. Other potential issues include infection, bleeding, and over-drainage [18].

In addition to complications related to the shunt itself, there's also a risk of abdominal complications with VP shunts, reported to be around 25%. These may include bowel obstruction, hernia, meningitis, and other issues related to the shunt tubing's placement in the abdominal cavity. It's essential to work closely with your healthcare team to monitor and manage any potential complications. Regular follow-up appointments and symptom monitoring can help ensure that any issues are identified and addressed promptly [19].

Our patient, who was asymptomatic, presented only with a VP shunt protruding from the anus. Culture results indicated the growth of Providencia stuartii, a gram-negative organism that can cause meningitis. For any patient presenting with abdominal symptoms, an evaluation for bowel perforation is crucial. The appropriate treatment varies depending on the presence of sepsis, perforation, peritonitis, or an intraperitoneal abscess.

In cases of simple bowel perforation without additional complications, a formal exploratory laparotomy may not be necessary. Instead, the shunt should be disconnected at the abdominal wall, and the lower end removed through the rectum. This can be done using colonoscopy, sigmoidoscopy, or proctoscopy to prevent contamination of the tract. The distal end of the VP shunt should not be pulled back into the peritoneal cavity. An external ventriculostomy should be established for at least three weeks, and the patient should be administered broad-spectrum antibiotics to prevent CSF infection. Once repeated CSF cultures return as sterile, a new VP shunt can be inserted on the opposite side. In more severe cases involving bowel perforation and peritonitis, an exploratory laparotomy is warranted. The shunt should be removed, the bowel wall thoroughly cleansed, and primary closure performed [20].

## Conclusions

The VP shunt functions by removing excess CSF from the brain's ventricles and transporting it to the peritoneal cavity, where the body can absorb and eliminate it. A valve is typically integrated into the shunt system to regulate the CSF's flow and avoid over-drainage or under-drainage. No doubt, one of the common procedures performed in neurosurgical practice is the placement of a VP shunt, but this procedure can result in several severe complications.

To prevent complications with a VP shunt, it is important for patients and caregivers to be aware of the signs and symptoms of shunt malfunction or infection, such as headaches, vomiting, seizures, meningitis, and changes in consciousness or behavior. Regular follow-up appointments with a healthcare provider are also important to monitor the shunt's function and address any concerns or complications that may arise.

## References

[1] Hack F, Oder A, Baumgartner C, Lomoschitz FM (2022). Intracardial migration of a ventriculoperitoneal shunt. BMJ Case Rep.

[2] Huang AP, Kuo LT, Lai DM, Yang SH, Kuo MF (2022). Antisiphon device: a review of existing mechanisms and clinical applications to prevent overdrainage in shunted hydrocephalic patients. Biomed J.

[3] Khizar A, Zahid S (1055052016150462022). Anal protrusion of peritoneal end of ventriculoperitoneal shunt and multiple brain abscesses: a case report with review of literature. Iran J Neurol.

[4] Trimmel NE, Podgoršak A, Oertel MF, Jucker S, Arras M, Schmid Daners M, Weisskopf M (2022). The sheep as a comprehensive animal model to investigate interdependent physiological pressure propagation and multiparameter influence on cerebrospinal fluid dynamics. Front Neurosci.

[5] Aspide R, Migliorino E, Pirina A (2022). Ventriculoatrial shunt under locoregional anesthesia: a technical note. World Neurosurg.

[6] Isaacs AM, Ball CG, Hamilton MG (2022). Neuronavigation and laparoscopy guided ventriculoperitoneal shunt insertion for the treatment of hydrocephalus. J Vis Exp.

[7] Muram S, Isaacs AM, Sader N, Holubkov R, Fong A, Conly J, Hamilton MG (2023). A standardized infection prevention bundle for reduction of CSF shunt infections in adult ventriculoperitoneal shunt surgery performed without antibiotic-impregnated catheters. J Neurosurg.

[8] Chimaliro S, Hara C, Kamalo P (2023). Mortality and complications 1 year after treatment of hydrocephalus with endoscopic third ventriculostomy and ventriculoperitoneal shunt in children at Queen Elizabeth Central Hospital, Malawi. Acta Neurochir (Wien).

[9] Gedde SJ, Feuer WJ, Lim KS, Barton K, Goyal S, Ahmed II, Brandt JD (2022). Postoperative complications in the primary tube versus trabeculectomy study during 5 years of follow-up. Ophthalmology.

[10] Pham C, Bennett I, Jithoo R (2017). Cryptococcal meningitis causing obstructive hydrocephalus in a patient on fingolimod. BMJ Case Rep.

[11] Ruspanah I, Hermanto Y, Taihuttu YM, Ruspanah A, Adam A, Imron A (2022). Implications of VP-shunt, sodium level, glucose level ratio and neurologic deficit as clinical outcome prognostic factor in adult meningitis tuberculosis with acute hydrocephalus in Dr Hasan Sadikin General Hospital. Bali Med J.

[12] Grewal SS, Jhawar SS, Gupta B, Bedi NK (2014). Silent bowel perforation with per anal protrusion of ventriculoperitoneal shunt. CHRISMED J Health Res.

[13] Guthe SP, Pravin S, Darade P, Velho V (2018). Silent migration of ventriculoperitoneal shunt per anus in a child: management and review of literature. Asian J Neurosurg.

[14] Park YS (2022). Treatment strategies and challenges to avoid cerebrospinal fluid shunting for pediatric hydrocephalus. Neurol Med Chir (Tokyo).

[15] Das S, Montemurro N, Ashfaq M (2022). Resolution of papilledema following ventriculoperitoneal shunt or endoscopic third ventriculostomy for obstructive hydrocephalus: a pilot study. Medicina (Kaunas).

[16] Lo TY, Myles LM, Minns RA (2003). Long-term risks and benefits of a separate CSF access device with ventriculoperitoneal shunting in childhood hydrocephalus. Dev Med Child Neurol.

[17] Ghritlaharey RK, Budhwani KS, Shrivastava DK, Gupta G, Kushwaha AS, Chanchlani R, Nanda M (2007). Trans-anal protrusion of ventriculo-peritoneal shunt catheter with silent bowel perforation: report of ten cases in children. Pediatr Surg Int.

[18] Fernandez B, Gautier A, Koumaré IB, Fabre JM, Coubes P, Poulen G (2022). Transcutaneous ventriculo-peritoneal shunt catheter extrusion with silent bowel perforation following digestive surgery: a case report. Br J Neurosurg.

[19] Harischandra LS, Sharma A, Chatterjee S (2019). Shunt migration in ventriculoperitoneal shunting: a comprehensive review of literature. Neurol India.

[20] Hai A, Rab AZ, Ghani I, Huda MF, Quadir AQ (2011). Perforation into gut by ventriculoperitoneal shunts: a report of two cases and review of the literature. J Indian Assoc Pediatr Surg.

